# Determining an infectious or autoimmune etiology in encephalitis

**DOI:** 10.1002/acn3.51608

**Published:** 2022-06-17

**Authors:** Hai Ethan Hoang, Jessica Robinson‐Papp, Lan Mu, Kiran T. Thakur, Jacqueline Sarah Gofshteyn, Carla Kim, Vivian Ssonko, Rachelle Dugue, Eileen Harrigan, Brittany Glassberg, Michael Harmon, Allison Navis, Mu Ji Hwang, Kerry Gao, Helena Yan, Nathalie Jette, Anusha K. Yeshokumar

**Affiliations:** ^1^ Weill Cornell Medical Center and New York Presbyterian Hospital New York New York USA; ^2^ Icahn School of Medicine at Mount Sinai Hospital New York New York USA; ^3^ Columbia University Irving Medical Center and New York Presbyterian Hospital New York New York USA

## Abstract

**Objectives:**

Early presentation and workup for acute infectious (IE) and autoimmune encephalitis (AE) are similar. This study aims to identify routine laboratory markers at presentation that are associated with IE or AE.

**Methods:**

This was a multi‐center retrospective study at three tertiary care hospitals in New York City analyzing demographic and clinical data from patients diagnosed with definitive encephalitis based on a confirmed pathogen and/or autoantibody and established criteria for clinical syndromes.

**Results:**

Three hundred and thirty‐three individuals with confirmed acute meningoencephalitis were included. An infectious‐nonbacterial (NB) pathogen was identified in 151/333 (45.40%), bacterial pathogen in 95/333 (28.50%), and autoantibody in 87/333 (26.10%). NB encephalitis was differentiated from AE by the presence of fever (NB 62.25%, AE 24.10%; *p* < 0.001), higher CSF white blood cell (WBC) (median 78 cells/μL, 8.00 cells/μL; *p* < 0.001), higher CSF protein (76.50 mg/dL, 40.90 mg/dL; *p* < 0.001), lower CSF glucose (58.00 mg/dL, 69.00 mg/dL; *p* < 0.001), lower serum WBC (7.80 cells/μL, 9.72 cells/μL; *p* < 0.050), higher erythrocyte sedimentation rate (19.50 mm/HR, 13.00 mm/HR; *p* < 0.05), higher C‐reactive protein (6.40 mg/L, 1.25 mg/L; *p* = 0.005), and lack of antinuclear antibody titers (>1:40; NB 11.54%, AE 32.73%; *p* < 0.001). CSF‐to‐serum WBC ratio was significantly higher in NB compared to AE (NB 11.3, AE 0.99; *p* < 0.001). From these findings, the association of presenting with fever, CSF WBC **≥**50 cells/μL, and CSF protein **≥**75 mg/dL was explored in ruling‐out AE. When all three criteria are present, an AE was found to be highly unlikely (sensitivity 92%, specificity 75%, negative predictive value 95%, and positive predictive value 64%).

**Interpretations:**

Specific paraclinical data at initial presentation may risk stratify which patients have an IE versus AE.

## Introduction

Encephalitis encompasses a set of central nervous system (CNS) diseases consisting of inflammation of the brain, manifesting with but not limited to decreased level of consciousness, behavioral changes, and seizures.^1^ Suspicion is supported by evidence of inflammation on cerebrospinal fluid (CSF) analysis or brain magnetic resonance imaging (MRI), and/or abnormalities on electroencephalography (EEG).^2^ The most common causes of encephalitis are due to infectious pathogens and antibody‐mediated autoimmune dysfunction.^2^ However, the broad differential includes neoplastic, vasculitis, epileptic, toxic, metabolic, and drug‐induced causes.^3^

It is important to distinguish between an infectious or autoimmune etiology of acute encephalitis early as possible because their definitive treatments and ultimate prognosis differ greatly.^2^ Despite similar prevalence (13.2/100,000 vs. 11.6/100,000, respectively) and median age (43.0 years [range 2.0–74.0] versus 43.0 years [0.0–91.0], respectively) for autoimmune and infectious encephalitis (IE),^4^ treatment for an autoimmune etiology is rarely initiated on the first few days of a first‐time presentation.^5^ One explanation is the widely accepted paradigm that an infection should be ruled out before initiating immunomodulatory treatment.

In this study, we conducted a multi‐site retrospective examination of patients data collected in the New York City Encephalitis Consortium, a cooperative of neurologists in the New York City area dedicated to studying encephalitis. Patients included were those admitted to one of three sites who ultimately received a definitive diagnosis of IE or autoimmune encephalitis (AE). The objective was to identify early clinical and laboratory data that may aid in the diagnosis of an IE or AE.

## Methods

This was a multi‐center retrospective study aiming to identify biomarkers associated with CNS inflammatory conditions of infectious or autoimmune origin.

### Standard protocol approvals, registrations, and patient consents

The study was independently approved by the Institutional Review Board (IRB)/Ethics Committee at each respective site. Given the retrospective nature of this study, a waiver of consent was obtained from all three IRBs.

### Study population

This study included patients admitted to the Weill Cornell Medical Center, Mount Sinai Health System, or Columbia University Irving Medical Center, between 01 January 2010, and 31 December 2017 with a first‐time diagnosis of a CNS inflammatory disorder.

**Figure 1:**
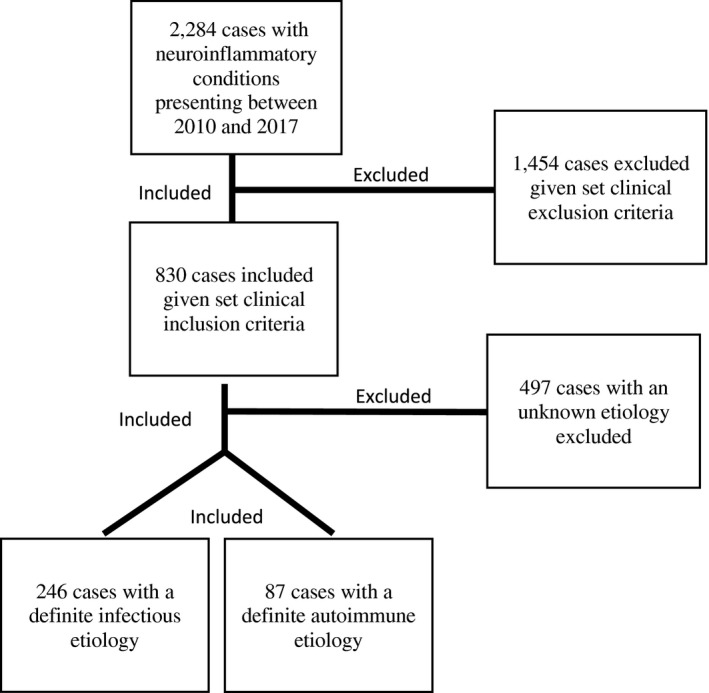
Flowchart of study population.

Figure 1 presents a flowchart of patients included in this study. Cases were identified according to pre‐specified ICD‐9‐CM codes encompassing suspected and known IE and AE, meningitis, meningoencephalitis, encephalomyelitis, and vasculitis (hereafter referred to broadly as “infectious encephalitis” or “autoimmune encephalitis”). ICD‐9‐CM was verified by detailed electronic medical record (EMR) review in all cases to ensure they met defined diagnostic criteria.^6^ To confirm clinical criteria, all EMRs were retrospectively reviewed at each site by at least two clinicians (H. E. H., A. N., A. K. Y., K. T. T., R. D., E. H., J. S. G., and M. J. H.). To diminish risk for bias, each case was reviewed by two reviewers who were both blinded to any pre‐specified hypotheses when reviewing the data. An encephalitis was diagnosed based on established case definitions.^7^ Cases were included if they were 1 month to 95 years old with a new‐onset encephalopathy and found to have a definitive pathogen or autoantibody associated with their encephalitis. Cases were excluded if there was a preceding diagnosis of IE or AE or if the patient presented with a neuroinflammatory myelopathy without evidence of an encephalitis. No cases were included if both an infectious and autoimmune pathogen were identified. Cases were excluded if a patient's EMR had significant amount of data missing (such as cases where the evaluation and diagnosis was fully completed at an outside hospital before being transferred for additional care but limited clinical documentation). A comprehensive list of inclusion and exclusion criteria can be provided if requested. Patients were broadly categorized as having an infectious or autoimmune etiology. Within the infectious cohort, either bacterial or nonbacterial (viral and fungal) etiology was denoted. Categorizing a patient as infectious or autoimmune required: (1) evidence of the etiologic agent (infectious pathogen or autoantibody) in serum, CSF, and/or pathology sample, and (2) a clinical syndrome that has been described in association with the etiologic agent in the CNS.^1^, ^2^, ^8^, ^9^, ^10^, ^11^, ^12^ Patients diagnosed with an autoimmune etiology must have additionally met Graus et al. 2016 criteria for definitive autoimmune limbic encephalitis or anti‐N‐methyl‐d‐aspartic acid (NMDA) encephalitis.^1^ Any disagreements related to diagnosis for IE and AE were separately reviewed and adjudicated by the site PI for each institution (A. K. Y., K. T. T., and J. S. G.). The case was included if there was sufficient documentation to demonstrate a clinical presentation and workup consistent with the determined etiology.

### Data collection

After initial review, data were extracted from the EMR by study team members using a structured data collection form for those patients meeting study inclusion criteria. We defined early clinical data points as those that can often be obtained and usually result within the first 48 h of the patient's initial hospitalization for encephalitis. These included: subjective/objective evidence of fever during the 1 week prior to admission or within the first 48 h of admission; CSF studies (CSF white blood cell (WBC) count and differential, glucose, protein, oligoclonal bands (OCBs), Meningitis/Encephalitis multiplex polymerase chain reaction panel^13^); serum studies (WBC count and differential, antinuclear antibodies (ANA), extractable nuclear antigen (ENA), anti‐neutrophilic cytoplasmic antibody (ANCA), rheumatoid factor (RF), erythrocyte sedimentation rate (ESR), C‐reactive protein (CRP), and lactate); and radiographic, and electroencephalographic data. Additional CSF data including lactate were not included since it was not consistently evaluated at our institutions. Confirmatory data including serum and CSF paraneoplastic/autoimmune antibodies, antigen, individual PCRs, cultures, serologies, and brain biopsies were also collected, but were not considered in the analysis given that the turnaround time for these tests average 14 or more days and not available at most institutions. All collected data were reviewed by the neurology service in charge of the patient at the time. Final MRI and EEG reports were determined by neuroradiologists and epileptologists at each institution, respectfully. Because of the variable workup for CNS inflammatory diseases across patients and the retrospective nature of this study, some data points of interest were not available for all patients.

### Statistical analysis

Descriptive analyses, including frequencies and medians with minimum and maximum ranges, were performed for all demographic and clinical variables. Categorical variables were compared in univariate analyses via frequencies and percentages with chi‐square analyses, while continuous variables were evaluated via medians with minimum and maximum ranges via Wilcoxon rank‐sum test. Ratios in the CSF and serum for WBC, neutrophils, lymphocytes, and monocytes were calculated.

Primary analyses were performed to identify variables associated with IE or AE. These were repeated comparing only infectious‐nonbacterial and autoimmune groups. Skewness of continuous variables was reduced using square‐root transformation. Two sets of multivariate regression models (four models total) were developed to identify independent variables associated with infectious versus immune and infectious‐nonbacterial versus AE. Sex, age group, race, fever, CSF WBC, CSF protein, CSF glucose, and serum WBC were entered into all models. The final models comprised variables selected by backward stepwise selection with a stopping criterion determined by the Akaike information criterion. Odds ratios (OR) with 95% confidence intervals (CI) were interpreted for all continuous and categorical variables. Adjusted *R* ^2^ was used to evaluate the goodness of fit of the final models. The first set of models included variables felt to be clinically significant and with data available for more than 2/3 of the cohorts (sex, age group, race, fever, CSF WBC, CSF protein, CSF glucose, and serum WBC). In a second set of exploratory models, we also included variables with larger amounts of missing data (CSF OCBs, ESR, CRP, ANA, MRIs with contrast‐enhancement, and MRI fluid‐attenuated inversion recovery (FLAIR) abnormalities). In this second analysis, missing data were imputed using multiple imputation by chained equations (MICE), under assumption of missing at random. The pooled OR and 95% CI, as well as adjusted *R* ^2^ with Fisher’s Z transformation, were estimated from final logistic regression models produced with backward elimination on the imputed datasets.

Finally, we used the results of the primary analyses to identify the variables which best differentiated autoimmune from infectious etiology to develop a clinically useful set of criteria to guide clinical decision‐making. We applied the screening tool to our current dataset to analyze the sensitivity, specificity, negative predictive value, positive predictive value when specific criterion was met.

A *p* < 0.05 was deemed significant. All analyses were performed in SPSS Statistics version 24 (Chicago, IL).

### Data availability

Anonymized data not published within this article will be made available by request from any qualified investigator.

## RESULTS

### Characteristics present in IE and AE

#### Patient demographics

Of 2284 patients initially identified based on ICD‐9‐CM codes, 1951 were excluded based on study criteria. The remaining 333 patients were identified as being diagnosed with a pathogen‐confirmed or autoantibody‐detected encephalitis. Participants were 50.45% female, and the median age was 40 years (range 0.10–95 years). The most reported races were White (*n* = 136; 40.84%) and Black (*n* = 61; 18.30%); Hispanic/Latin ethnicity was not consistently reported. Table 1 provides a more detailed description of the total population in the study including details for patients in the infectious group, infectious‐nonbacterial subgroup, and autoimmune group.

**Table 1:** Demographic characteristics in infectious and autoimmune encephalitis.

Demographics					*p*‐value	*p*‐value
	Total *n* = 333	Infectious encephalitis^1^ *n* = 246	Infectious‐nonbacterial *n* = 151	Autoimmune encephalitis *n* = 87	Infectious versus autoimmune	Infectious‐nonbacterial versus autoimmune
Sex					0.001	<0.001
Male	49.5%	54.9%	58.9%	34.5%		
Female	50.5%	45.1%	41.1%	65.5%		
Age^2^	40 (0–95)	40 (0–95)	41 (0–95)	37 (3–86)	0.097	0.246
0–4^3^	37 (11.1%)	33 (13.4%)	15 (9.9%)	4 (4.6%)		
5–19^3^	40 (12.0%)	29 (11.8%)	18 (11.9%)	11 (12.6%)		
20–44^3^	110 (33.0%)	78 (31.7%)	50 (33.11%)	32 (36.8%)		
45–64^3^	82 (24.6%)	60 (24.4%)	33 (21.9%)	22 (25.3%)		
>64^3^	64 (19.2%)	46 (18.7%)	35 (23.2%)	18 (20.7%)		
Race					0.015	0.021
Asian/Native American, Other^3^	92 (27.6%)	60 (24.4%)	35 (23.2%)	32 (36.8%)		
Black/Afr. American^3^	61 (18.3%)	42 (17.1%)	26 (17.2%)	19 (21.8%)		
White^3^	136 (40.8%)	105 (42.7%)	62 (41.1%)	31 (35.6%)		
Unknown^3^	44 (13.2%)	39 (15.9%)	28 (18.5%)	5 (5.7%)		

^1^ Includes infectious‐nonbacterial, bacterial, and fungal.

#### Disease etiology

Two hundred and forty‐six patients (73.87%) were identified as having an IE (bacterial, infectious‐nonbacterial) while 87 (26.13%) individuals were identified as having an AE (Table 2). Table 2 lists pathogens with greater than five cases in each inflammatory cohort. *Streptococcus* (*n* = 55; 22.40%) and anti‐NMDA receptor antibody (*n* = 35; 40.20%) were the most frequently found etiologies in IE and AE, respectively. In the infectious‐nonbacterial subgroup, herpes simplex virus (HSV) (*n* = 39; 25.83%) was the infectious pathogen most often identified. Although, the authors recognize that voltage‐gated potassium channelopathies (VGKC) are now subclassified as leucine‐rich glioma‐inactivated 1 (LGI1) or contactin‐associated protein 1 (CASPR), the timeline of this study (2010–2017) included a significant period where LGI1 and CASPR were not readily commercially available to test.^14^ All authors agreed that combining the LGI1 and CASPR cases under the heading VGKC was the most comprehensive way to include all cases without creating three groups (VGKC, LGI1, and CASPR) that would have overlap. Additionally, the retrospective nature of this study prevented retesting and subclassification of cases initially labeled as VGKC‐mediated.

**Table 2:** Summary of pathogen and autoantibodies found in our study.

Infectious encephalitis						Autoimmune encephalitis	
Bacterial		Infectious‐nonbacterial				*n* = 87	
*n* = 95		Viral *n* = 132		Fungal *n* = 19			
*Streptococcus*	55	Herpes Simplex Virus	39	*Cryptococcus*	13	NMDA	35
Lyme	10	Varicella Zoster Virus	36			NMO	12
Listeria	6	Enterovirus	33			VGKC (LGI1 and CASPR)	11
		Human Herpes Virus 6	6			GAD 65	6

NMDA, N‐methyl‐d‐aspartate; NMO, neuromyelitis optica; VGKC, voltage‐gated potassium channel; LGI1, leucine‐rich glioma‐inactivated 1; CASPR, contactin‐associated protein‐like 2; GAD 65, glutamic acid decarboxylate 65.

#### Clinical presentation with fever

The presence of fever within the 1 week prior to or 48 h after initial presentation was reported in 184 (55.30%) patients (Table 3). Subjective and objective fever were more likely in infectious than AE patients. Similarly, infectious‐nonbacterial encephalitis patients were more likely to present with a fever compared to AE patients.

**Table 3:** Clinical presentation, CSF, and serum characteristics in infectious and autoimmune encephalitis.

	Total	Infectious^1^ encephalitis	Infectious‐nonbacterial	Autoimmune encephalitis	*p*‐value	*p*‐value
					Infectious versus autoimmune	Infectious‐nonbacterial versus autoimmune
Clinical presentation	*n* = 333	*n* = 246	*n* = 151	*n* = 87		
Presence of fever^2^	184 (55.3%)	163 (66.3%)	94 (62.3%)	21 (24.8%)	<0.001	<0.001
CSF studies	*n* = 296	*n* = 223	*n* = 139	*n* = 73		
WBC (cells/μL)^3^	50.0 (0–16,150)	100.0 (0–16,150)	78.0 (0–2184)	8.0 (0–300)	<0.001	<0.001
Neutrophils (%)^3^ ^,^ ^4^	10.0 (0–98)	13.5 (0–98)	7.0 (0–97)	2.0 (0–22)	<0.001	0.017
Lymphocytes (%)^3^ ^,^ ^4^	74.5 (0–100)	62.0 (0–100)	77.0 (0–100)	92.0 (1–100)	<0.001	<0.001
Monocytes (%)^3^ ^,^ ^4^	8.0 (0–84)	8.0 (0–84)	9.0 (0–84)	6.0 (0–77)	0.306	0.166
Eosinophils (%)^3^ ^,^ ^4^	0.0 (0–21)	0.0 (0–7)	0.0 (0–7)	0.0 (0–21)	0.127	0.044
Protein (mg/dL)^3^	76.5 (13–5001)	97.0 (13–5001)	76.5 (13–1123)	40.9 (14–171)	<0.001	<0.001
Glucose (mg/dL)^3^ ^,^ ^4^	5.8 (0–221)	55.0 (0–221)	58.0 (14–221)	69.0 (24–160)	<0.001	0.001
CSF‐specific OCBs^4^	26/75 (34.7%)	9/25 (25.0%)	7/27 (25.9%)	17/39 (43.6%)	0.091	0.142
Serum studies	*n* = 327	*n* = 241	*n* = 147	*n* = 86		
WBC (cells/μL)^2^	9.1 (1.2–38)	9.0 (1.2–38)	7.8 (1.2–34.3)	9.7 (2.7–21.3)	0.98	0.02
Neutrophils (%)^2^ ^,^ ^4^	72.2 (16–95)	73.0 (16–95)	69.7 (16–95)	70.7 (26–95)	0.133	0.958
Lymphocytes (%)^2^ ^,^ ^4^	16.0 (1–64)	15.0 (1–61)	17.0 (1–61)	19.1 (1–64)	0.028	0.522
Monocytes (%)^2^ ^,^ ^4^	7.1 (0–25)	7.3 (0–20)	8.0 (1–20)	7.0 (1–25)	0.988	0.094
Basophils (%)^2^ ^,^ ^4^	0.1 (0–3.5)	0.1 (0–3.5)	0.2 (0–3.5)	0.0 (0–2.4)	0.417	0.231
Eosinophils (%)^2^ ^,^ ^4^	0.6 (0–18.9)	0.3 (0–10)	0.6 (0–8)	1.1 (0–18.9)	<0.001	0.006
ESR (mm/HR)^2^ ^,^ ^4^	20.0 (0–131)	27.0 (1–131)	19.5 (1–131)	13.0 (0–120)	<0.001	0.035
CRP (mg/L)^2^ ^,^ ^4^	6.9 (0.1–362)	11.3 (0.1–362)	6.4 (0.2–236.4)	1.3 (0.1–145)	<0.001	0.005
Lactate (mmol/L)^2^ ^,^ ^4^	1.5 (0.6–335)	1.5 (0.6–15.7)	1.4 (0.6–15.7)	1.2 (0.6–335)	0.049	0.285
ANA >1:80^4^	21/90 (23.3%)	3/35 (8.6%)	3/26 (11.5%)	18/55 (32.7%)	<0.001	<0.001
ENA positivity^4^	6/48 (12.5%)	0/16 (0%)	0/13 (0%)	6/32 (18.8%)	<0.001	<0.001
ANCA positivity^4^	4/52 (7.7%)	3/19 (15.8%)	2/12 (16.7%)	1/33 (3.0%)	<0.001	<0.001
RF positivity^4^	3/49 (6.1%)	2/17 (11.8%)	2/13 (15.4%)	(1/32) 3.1%	<0.001	<0.001

WBC, white blood cell; CSF, cerebrospinal fluid; OCB, oligoclonal bands; ESR, erythrocyte sedimentation rate; CRP, c‐reactive protein; ANA, antinuclear antibody; ENA, extractable nuclear antibody; ANCA, anti‐neutrophilic cytoplasmic antibody; RF, rheumatoid factor.

^1^ Includes infectious‐nonbacterial, bacterial, and fungal.

^2^ Values are *n* (%).

^3^ Values are median (minimum, maximum).

^4^ Results not available for all patients: total *n* are identified for the following. CSF neutrophils *n* = 217, lymphocytes *n* = 244, monocyte *n* = 239, eosinophils *n* = 112, basophils *n* = 37, glucose *n* = 295; serum/plasma OCB *n* = 75, neutrophils *n* = 299, lymphocytes *n* = 298, monocytes *n* = 298, eosinophils *n* = 246, ESR *n* = 150; CRP *n* = 162, lactate *n* = 190, ANA *n* = 90, ENA *n* = 48, ANCA *n* = 52, RF *n* = 49.

### CSF characteristics

CSF WBC median across all patients was 50.0 cells/μL (Table 3). CSF pleocytosis (defined as CSF WBC >5 cells/μL) was more likely to be seen in patients with IE compared to AE. Similarly, patients with infectious‐nonbacterial encephalitis also had a more elevated CSF WBC count compared to AE patients. CSF protein was significantly elevated (normal CSF protein 15–45 mg/dL) in both the infectious group and infectious‐nonbacterial encephalitis subgroup compared to AE patients. In the subset of patients in whom CSF OCBs were measured (*n* = 75), no difference was seen in the presence of unique CSF OCBs between the infectious compared to the AE. Similarly, there was no difference between the infectious‐nonbacterial and AE cohort. In our study, of the nine infectious patients with unique CSF OCBs, three were found to have Herpes Simplex Virus and two Varicella Zoster Virus. The remaining four patients were found to have either Lyme, *Streptococcus*, *Cryptococcus*, or Human Herpes Virus 6.

### Serum characteristics

The median serum WBC across all patients was 9.1 cells/μL (Table 3). There was no difference in serum WBC between IE and AE groups. However, AE patients did have a higher serum WBC (normal serum WBC 4.5–11.0 cells/μL) compared to infectious‐nonbacterial encephalitis patients. The inflammatory markers ESR (normal serum ESR <15 mm/HR) and CRP (normal serum CRP <5 mg/L) were abnormally elevated in IE patients compared to AE. Similar findings were seen when comparing ESR and CRP in infectious‐nonbacterial and AE patients. Serum lactate (normal serum lactate <1.6 mmol/L) was higher in infectious compared to AE patients, but the difference in lactate levels were not seen in the infectious‐nonbacterial encephalitis subset of patients. Rheumatologic markers including ANA, ENA, ANCA, and RF were more frequently abnormal in autoimmune compared to infectious and infectious‐nonbacterial subgroup of encephalitis patients.

### MRI and EEG characteristics

The presence of contrast‐enhancement on brain MRI was more frequently noted in infectious compared to AE patients, but no significant difference was seen when comparing infectious‐nonbacterial and AE patients (Table 4). The presence of FLAIR abnormalities on brain MRI, any EEG abnormalities (including abnormalities like moderate generalized slowing and epileptiform activity), and solely EEGs with epileptiform activity were not different in the IE and AE groups, respectively.

**Table 4:** MRI and EEG characteristics in infectious and autoimmune encephalitis.

	Total	Infectious encephalitis^1^	Infectious‐nonbacterial	Autoimmune encephalitis	*p*‐value Infectious versus autoimmune	*p*‐value Infectious‐nonbacterial versus autoimmune
MRI	*n* = 216	*n* = 140	*n* = 77	*n* = 77		
Contrast‐enhancing	34.9%	42.0%	24.7%	22.1%	0.003	0.709
FLAIR‐abnormalities	55.6%	53.6%	54.5%	59.2%	0.426	0.56
EEG	*n* = 150	*n* = 92	*n* = 51	*n* = 58		
Epileptic activity	29.3%	27.2%	33.3%	32.8%	0.464	0.949
EEG abnormalities^2^	90.6%	90.1%	91.8%	91.4%	0.796	0.932

MRI, magnetic resonance imaging; FLAIR, fluid‐attenuated inversion recovery; EEG, electroencephalography.

^1^ Includes infectious‐nonbacterial, bacterial, and fungal.

^2^ Abnormal EEGs include EEGs in which epileptic activities were noted.

### CSF and serum WBC ratios

**Figure 2:**
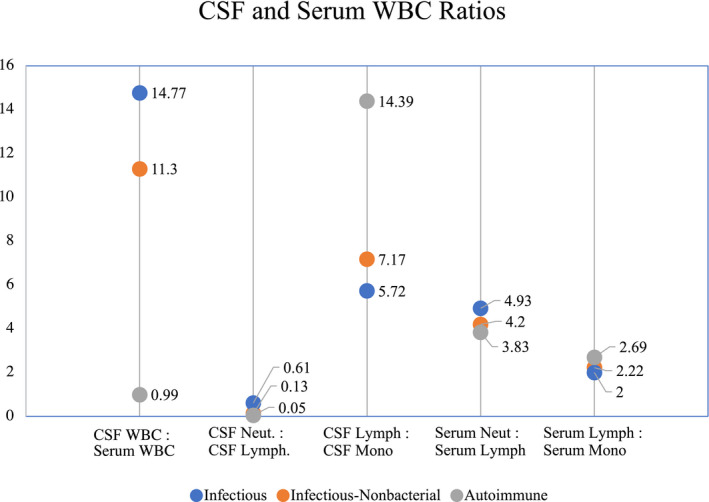
CSF and serum WBC ratios in infectious and autoimmune encephalitis. Values represents median. CSF WBC, serum WBC *p* < 0.05; CSF neutrophils, CSF lymphocytes *p* < 0.05; CSF lymphocytes, CSF monocytes *p* < 0.05; Serum neutrophils, serum lymphocytes *p* > 0.05; Serum lymphocytes, serum monocytes *p* > 0.05; WBC, white blood cell.

The median CSF WBC‐to‐serum WBC ratio was significantly higher in infectious compared to AE (Fig. 2). A similar trend was noted in the infectious‐nonbacterial compared to autoimmune group, respectively. When looking at the differential in CSF WBC, CSF neutrophil‐to‐lymphocyte ratios were significantly higher when comparing both infectious and infectious‐nonbacterial encephalitis to AE. A difference was also noted in the serum neutrophil‐to‐lymphocyte ratio for infectious compared to AE, but not infectious‐nonbacterial compared to AE. CSF lymphocyte‐to‐monocyte ratios were significantly higher when comparing both infectious and infectious‐nonbacterial encephalitis to AE. Like the serum neutrophil‐to‐lymphocyte ratio, a difference was noted in the serum lymphocyte‐to‐monocyte ratio for infectious compared to AE, but not infectious‐nonbacterial compared to AE.

### Multivariate analysis: laboratory markers associated with AE

We developed two sets of multivariate regression models (total of four models) to identify independent variables associated with infectious versus autoimmune and infectious‐nonbacterial versus AE (Table 5). The first model included variables felt to be clinically significant which had data available for more than 2/3 of the study population. A second exploratory model was developed to include (1) all variables with more than 2/3 data and (2) all variables felt to be clinically significant despite having more than 1/3 missing data. In this second model, all data were imputed for variables with missing data. In comparing IE and AE (model 1), higher serum WBC, lower CSF WBC, lower CSF protein, lack of fever, and age 12–29 years (compared to >65 years) were associated with an autoimmune CNS etiology. In the imputed model 2, the presence of FLAIR abnormalities and lack of contrast‐enhancement were also associated with an autoimmune CNS process. In comparing infectious‐nonbacterial versus AE (model 3), higher serum WBC, lower CSF WBC, lack of fever, and age 12–29 years (compared to >65 years) were associated with an AE etiology. In the imputed model 4, the presence of MRI FLAIR changes and a lower CRP were also associated with AE.

**Table 5:** Characteristics predictive of autoimmune encephalitis.

Infectious encephalitis versus autoimmune encephalitis^1^	Model 1^2^		Model 2^3^	
	Odds ratio (CI)	*p*‐value	Odds ratio (CI)	*p*‐value
	*r* ^2^ = 0.36		*r* ^2^ = 0.42	
Presence of fever	0.36 (0.17–0.77)	0.008	0.21 (0.08–0.54)	<0.002
Elevated Serum WBC^3^	1.87 (1.11–3.16)	0.019	1.94 (1.08–3.48)	0.031
Elevated CSF WBC^3^	0.88 (0.82–0.95)	<0.001	–	–
Elevated CSF protein^3^	0.80 (0.70–0.92)	0.002	–	–
Age 12–29 years^4^	3.33 (1.17–9.49)	0.024	–	–
MRI FLAIR abnormalities	–	–	2.80 (1.13–6.89)	0.029
MRI contrast enhancing	–	–	0.30 (0.11–0.82)	0.024

Infectious‐nonbacterial encephalitis versus autoimmune encephalitis^5^	Model 3^2^		Model 4^3^	
	Odds ratio (CI)	*p*‐value	Odds ratio (CI)	*p*‐value
	*r* ^2^ = 0.35		*r* ^2^ = 0.51	
Presence of fever	0.30 (0.13–0.69)	0.005	0.31 (0.12–0.81)	0.018
Elevated serum WBC^3^	2.45 (1.37–4.39)	0.003	3.30 (1.44–7.52)	0.008
Elevated CSF WBC^3^	0.86 (0.79–0.93)	< 0.001	0.84 (0.75–0.95)	0.006
Age 12–29 years^4^	4.72 (1.49–14.93)	0.008	–	–
Elevated CRP^3^	–	–	0.77 (0.60–0.98)	0.045
MRI FLAIR abnormalities	–	–	3.38 (1.10–10.39)	0.040

WBC, white blood cell; CSF, cerebrospinal fluid; MRI, magnetic resonance imaging; FLAIR, fluid attenuated inversion recovery; CRP, C‐reactive protein.

^1^ Reference group = infectious.

^2^ Included as candidate variables that were deemed clinically significant and had >2/3 of cases were available.

^3^ Used imputed dataset; all variables were candidates for inclusion. Continuous variables were square‐root transformed.

^4^ Each age cohorts were compared to reference age cohort of individuals aged 65+ years.

^5^ Reference group = infectious‐nonbacterial.

### Diagnostic utility of combining routine markers: determining etiology in encephalitis (DEE) score

Using the results of the multivariable models to enhance the clinical applicability of our findings, we identified three variables that best discriminated an infectious from an autoimmune CNS etiology and sought to explore their cumulative predictive value for a diagnosis of AE. Two‐hundred ninety patients (87.08% of all patients in our study) had all three data points available for this exploratory analysis. These features were explored: fever (present vs. absent), CSF WBCs of ≥50 cells/μL, and CSF protein of ≥75 mg/dL (Table 6).

**Table 6:** Determining etiology in encephalitis score (DEE) score.

Patient characteristics	Score points
Presence of fever	1
CSF WBC ≥50 cells/μL	1
CSF protein ≥75 mg/dL	1

Number of criteria met	Patients with infectious etiology	Patients with infectious‐nonbacterial etiology	Patients with autoimmune etiology
0	36%	33%	64%
1	75%	58%	25%
2	93%	86%	7%
3	95%	90%	5%

WBC, white blood cell; CSF, cerebrospinal fluid.

In this group, an infectious etiology was found in 95% of patients presenting with all three criteria and in 93% of patients presenting with two out of three. When applying the score to patients with infectious‐nonbacterial encephalitis (90% of patients with infectious‐nonbacterial encephalitis presented with all three criteria, and 86% of patients presented with two criteria). Therefore, the presence of two or three of these criteria suggested that an autoimmune etiology was unlikely. However, patients presenting with either none or only one of these criteria were more diagnostically mixed (i.e., among patients meeting none of the criteria, 36% were infectious, and among patients meeting one criterion, 75% were infectious). When two criteria were present, the negative predictive value was 94%, positive predictive value was 63%, sensitivity was 83%, and specificity was 84%, suggesting strength in the model for ruling out AE. When all three criteria were present, the negative predictive value was 95%, positive predictive value was 64%, sensitivity was 92%, and specificity was 75%, again suggesting strength in ruling out AE.

## Discussion

Our study aims to characterize the patterns seen in CNS inflammatory diseases and develop a model predictive of disease etiology based on clinical biomarkers that are routinely obtained as part of clinical care. Given the rarity of these diseases, we had a large sample size of definitive cases and were able to develop a simple predictive model for ruling out AE. Prior studies have characterized the clinical profile of IE and AE, but these studies had (1) a smaller study of 95 patients,^15^ (2) evaluated infectious or autoimmune encephalitis biomarkers in isolation,^10^, ^11^ or (3) examined a more limited number of laboratory markers (e.g., serum ANA, TPO, CSF profile [cell count, protein, glucose, and OCB], and brain MRI, but no serum WBC, inflammatory markers [ESR, CRP, lactate], or EEG data).^16^ One study examined patients with IE or AE but did not find any significant differences between the groups in serum complete blood count, basic metabolic panel, or CSF cell count and protein.^15^ The California Encephalitis Project^10^ and the Infectious Disease Society of America^11^ have characterized the clinical profile of IE. In the former, the authors reported CSF WBC was elevated in infectious compared to noninfectious etiologies. Noninfectious causes were presumed to be autoimmune, but the commercial use of autoantibody panels had not yet been developed. Another study found ANA and thyroid antibodies titers to be elevated in probable and definite AE as compared to a disease‐free population.^16^

Our results identify routinely obtained clinical biomarkers that when present make an autoimmune CNS etiology less likely. Infectious CNS etiology in patients was associated with: the presence of fever (defined as the subjective/objective evidence of fever during the week prior to admission or within the first 48 h of admission); markedly elevated CSF WBC and protein; higher inflammatory markers including ESR, CRP, and lactate; and the presence of contrast‐enhancement on brain MRI. The presence of EEG abnormalities (including the spectrum from generalized slowing to epileptiform activity) were not unique to both groups. The presence of OCBs antibodies in the CSF was not unique to AE. This may in part be explained by the fact that OCBs and IgG antibodies are typically checked early in the diagnostic process, and OCBs and intrathecal immunoglobulin G (IgG) synthesis may occur later in acute CNS autoimmune diseases.^17^, ^18^ Additionally, since elevated CSF IgG synthesis reflects local production and/or breach in the blood–brain barrier, CNS infections including Herpes Simplex Virus and Varicella Zoster Virus^19^ can also result in unique CSF OCBs as was seen in our patients.

Our study was also able to identify the triad of (1) the presence or history of fever, (2) CSF WBC ≥50 cells/μL, (3) and CSF protein ≥75 mg/dL as potentially helpful parameters to rule out AE. When all three criteria were present in a patient, the negative predictive value was 95%, suggesting utility in ruling out AE. To compare, a smaller study suggested that a CSF WBC cutoff ≤36 cells/μL had a sensitivity of 75% and specificity of 87.5% for diagnosing AE.^17^ While a more accurate scoring system could be generated with the inclusion of criteria such as autoantibody panels, the strength of our proposed scoring system is its reliance on variables that are typically processed in‐house at many institutions making it a widely implementable tool. This screen is also noteworthy in its ability to be used early in a patient's admission to prioritize certain infectious or autoimmune tests as these variables take <24 h to be processed at most institutions. With regards to AE, few diagnostic scoring systems exist. The APE2 score was developed to look specifically at predictors of autoimmunity in epilepsy.^20^ Other scoring systems such as RITE2^20^ and CASE^21^ focus either on the predictors of favorable outcomes in autoimmune epilepsy or the clinical severity of AE, respectively. However, these scores are only utilized for prognostication, and not during diagnosis.

Since their clinical and diagnostic profiles at first presentation may appear similar, a second question our study aimed to explore was the differences specifically between the infectious‐nonbacterial subgroup (patients with IE etiologies that were not bacterial in origin) and the autoimmune CNS population.^22^ While the basic CSF profile of patients with bacterial and infectious‐nonbacterial encephalitis are starkly different, both infectious‐nonbacterial and AE can have mild elevations in CSF WBC and protein. Our study demonstrated that most of our patients diagnosed with autoimmune rather than infectious‐nonbacterial encephalitis presented without evidence of fever, had normal CSF WBC and protein, and normal serum ESR and CRP. EEG was not helpful in distinguishing the two groups. An interesting finding in our study was that a higher median serum WBC (but still less than the threshold to be considered abnormally elevated) was present in autoimmune compared to infectious‐nonbacterial encephalitis. However, this trend was not seen when comparing the infectious (e.g., bacterial, and infectious‐nonbacterial) to AE groups. One theory for the lower WBC in the blood of infectious‐nonbacterial encephalitis patients is potential disruption of bone marrow hematopoiesis and subsequent reduced progenitor cell lineages due to infectious‐nonbacterial infection.^15^

Our study was able to identify higher CSF‐to‐serum WBC ratios in both infectious and infectious‐nonbacterial encephalitis compared to AE. To our knowledge, no study has compared the WBC ratios in IE and AE. Prior research has demonstrated that ratios of serum and CSF WBC neutrophils‐to‐lymphocytes are elevated in IE^23^ and AE.^24^ CSF neutrophil‐to‐lymphocyte ratios were higher in infectious and infectious‐nonbacterial encephalitis compared to AE, but CSF lymphocyte‐to‐monocyte ratios were lower. Further research is needed to determine whether these ratios are beneficial in a clinical setting.

There are several limitations to our study, mostly pertaining to its retrospective nature and heterogeneous population. Due to the rarity of CNS inflammatory diseases, amassing a collection of definite diagnoses is inherently challenging and required participation from multiple hospitals. Approaches to diagnostic evaluation differed, and not all variables of interest were collected for each patient. A larger cohort and fewer missing data may have enabled multivariate analyses to detect more differences between the variables. It must also be noted that we limited our study to pathogen‐confirmed and autoantibody‐detected patients and did not include cases where a definitive diagnosis was not established because we did not want to risk misclassification since there are not strong level of evidence for diagnostic criteria for IE and AE. Thus, our study includes a specific sub‐population in which a definitive pathogen or autoantibody was identified because this was the cleanest cohort in which to initially identify factors that would enable the discrimination between AE and IE. We acknowledge that there are many cases that fall within the gray area between both types of encephalitides in clinical practice, and therefore, it is not yet clear how this study's findings may generalize to all cases in clinical practice. Furthermore, not all of our study patients found to have an infectious etiology were tested for autoimmune autoantibodies, especially if their clinical picture was explained by the infectious pathogen. Thus it is possible that some of our IE patients could have had a concomitant undiagnosed AE. One studied identified 7% of patients with a HSV encephalitis also had NMDA encephalitis.^25^ Although our model focuses on laboratory markers for clinical decision‐making, this is not intended to undervalue the unique features in a patient's history such as encephalopathy type (i.e., specific cognitive or psychiatric abnormalities), geographic location, seasonality, comorbid medical problems, and/or systemic exam findings on physical examination, which can aid in diagnosis. Age was not included in the DEE score because we wanted to incorporate variables in which we could create clear, biologically relevant cutoffs. Future studies will aim to test the scoring system in diverse populations including those with extremes of ages. We acknowledge these clinical features are helpful in suggesting an autoimmune or infectious etiology. However, given the retrospective nature of this study, their presence was not always clearly characterized in clinical documentation, and therefore they were not examined as predictive features in our model. Lastly, the data collected for the study population preceded the 2020 COVID‐19 pandemic and thus does not reflect any IE patients related to the COVID‐19 virus.

## Conclusion

Overall, this study's findings may be used as a guide in the diagnostic evaluation of =presumed CNS inflammatory cases in which clinical history alone is not sufficient to narrow the differential. Future work might include assessing the reproducibility of these findings in a retrospective cohort of patients with different geography, race, and age or in prospective studies to determine whether our models can be employed to improve time‐to‐treatment, hospitalization costs, and morbidity and mortality in patients with encephalitis.

## Author contributions

HEH, JRP, and AKY conceived and designed the study. HEH, KTT, CK, VS, RD, EH, BG, MH, AN, MJH, KG, HY, JG, NJ, and AKY collected the data. HEH, LM, JRP, AKY interpreted the data. HEH, JRP, AYK contributed to the writing of the manuscript. HEH, JRP, AYK have accessed and verified all the data in the study. All authors had full access to all the data in the study and had final responsibility for the decision to submit the publication.

## Conflict of Interest

Dr. Hai Hoang, Lan Mu MSc, Dr. Jessica Robinson‐Papp, Dr. Kiran Thakur, Carla Kim, Vivian Ssonko, Dr. Rachelle Dugue, Eileen Harrigan, Dr. Brittany Glassberg, Michael Harmon BS, Dr. Allison Navis, Dr. Mu Ji Hwang, Kerry Gao BA, Helena Yan BA, Dr. Jacqueline Sarah Gofshteyn, Dr. Nathalie Jette, and Dr. Anusha K. Yeshokumar reports no conflict of interests.
